# Resveratrol ameliorates atherosclerosis induced by high-fat diet and LPS in ApoE^−/−^ mice and inhibits the activation of CD4^+^ T cells

**DOI:** 10.1186/s12986-020-00461-z

**Published:** 2020-05-27

**Authors:** Liyu Zhou, Jun Long, Yuting Sun, Weikai Chen, Runze Qiu, Dongping Yuan

**Affiliations:** 1 Jiangsu Key Laboratory for Pharmacology and Safety Evaluation of Chinese Materia Medica, School of Pharmacy, Nanjing University of Chinese Medicine, Xianlin Dadao 138, Nanjing, 210023 Jiangsu People’s Republic of China; 2 Department of Clinical Pharmacology Laboratory, Nanjing First Hospital, Nanjing Medical University, Nanjing, 210006 People’s Republic of China

**Keywords:** Resveratrol, Atherosclerosis, CD4^+^ T cells, DNA methyltransferase

## Abstract

**Background:**

Atherosclerosis (AS), which characterized with the accumulation of lipids on the vessel wall, is the pathological basis of many cardiovascular diseases (CVD) and seriously threatens human health. Resveratrol (RES) has been reported to be benefit for AS treatment. This research aimed to observe the effects of RES on AS induced by high-fat diet (HFD) and LPS in ApoE^−/−^ mice and investigate the underlying mechanism.

**Methods:**

ApoE^−/−^ mice were fed with HFD companied with LPS to induce AS and RES was administrated for 20 weeks. Splenic CD4^+^ T cells were cultured and treated with anti-CD3/CD28 together with LPS, and RES was added. Serum lipids and the atherosclerotic areas of aortas were detected. The activation of CD4^+^ T cells were investigated both in vivo and in vitro and the expression of DNA methyltransferases (Dnmt) in CD4^+^ T cells were measured.

**Results:**

In vivo, administration of RES prevented HFD and LPS induced dysfunction of serum lipids including TC (total cholesterol), TG (triglyceride), LDL-C (low density lipoprotein cholesterol) and HDL-C (high density lipoprotein cholesterol), ameliorated the thickened coronary artery wall and decreased the areas of atherosclerotic lesion on aortas. Besides, RES decreased the number of CD4^+^ T cells in peripheral blood, decreased the expression of CD25 and CD44, but not affected the expression of L-selectin (CD62L). In vitro, RES decreased the expression of Ki67, CD25 and CD44 in CD4^+^ T cells. Moreover, RES increased the secretion of IL-2, IL-10 and TGF-β1, decreased IL-6. In addition, RES decreased both the mRNA and protein level of Dnmt1 and Dnmt3b in CD4^+^ T cells.

**Conclusion:**

These results indicated that RES ameliorated AS induced by HFD companied with LPS in ApoE^−/−^ mice, inhibited the proliferation and activation of CD4^+^ T cells and regulated the expression of Dnmt1 and Dnmt3b.

## Introduction

Atherosclerosis (AS) is a chronic inflammatory disease [1, 2]. AS induces the progression of cardiovascular diseases (CVD) such as coronary heart disease and cerebral infarction, seriously threatens human health [3]. Therefore, prevent the progression of AS is vital for keeping cardiovascular health.

The pathogenesis of AS is complex. Dyslipidemia is a risk factor for the progression of AS and keep the serum lipids in a normal range is an important way to prevent AS [4]. In addition, chronic inflammation, which accelerates the accumulation of immune cells on vessel wall, is another risk factor of AS [5]. Immune cells in atherosclerotic lesions producing mainly pro-inflammatory cytokines and accelerating the formation of an inflammatory microenvironment [2, 6]. CD4^+^ T cells are the most abundant T cells in atherosclerotic lesion and play important roles throughout the stages of atherogenesis [7]. CD4^+^ T cells as an important component in adaptive immune responses, powerfully regulates the inflammatory process [8, 9]. Naïve CD4^+^ T cells highly express of L-selectin (CD62L), and CD62L was down-regulated when CD4^+^ T cells were activated under inflammatory stimulation [10]. Moreover, CD25 and CD44 are activation biomarkers of CD4^+^ T cells and are potently induced after the activation [11]. Activated CD4^+^ T cells further activate the immune response, increase the secretion of pro-inflammatory cytokines like interleukin-6 (IL-6), and decrease IL-10 and transforming growth factor-β1 (TGF-β1) [12–14]. The activation of CD4^+^ T cells is an important process in the inflammation progression in AS and prevent the activation of CD4^+^ T cells would be expected to prevent inflammation and ameliorate AS [15].

Resveratrol (RES), a natural polyphenol presented in grapes, mulberries, peanuts, rhubarb, and in several other plants [16, 17], is a potential food ingredient to prevent CVD. RES has been reported to prevent AS progression in both mice and patients [18, 19] and the mechanisms refer to anti-oxidant, anti-platelet or anti-inflammatory [20]. RES protect AS in multiple ways, but the exact mechanism still unclarified and under discussion. It has been reported that RES regulates the immune response of CD4^+^ T cells by metabolic reprogramming, and inhibits CD4^+^ T cells activation by enhancing the expression of SIRT1 [21, 22]. RES regulates CD4^+^ T cells activation via multiple mechanisms and regulates CD4^+^ T cells mediated inflammation. Moreover, RES has been reported as epigenetically active agents which function in regulating DNA methyltransferases (Dnmt) expression [23, 24]. DNA methylation is one of the best characterized epigenetic modifications and was mediated by Dnmt including Dnmt1, Dnmt3a and Dnmt3b [25]. Abnormal expression of Dnmt related to the dysfunctional cellular activities [26], and the changed expression of Dnmt is a potential mechanism for regulating CD4^+^ T cells activities. But whether RES regulates the expression of Dnmt in CD4^+^ T cells remains unknown.

This research aimed to investigated the effects of RES on ameliorating AS induced by HFD and LPS in ApoE^−/−^ mice, inhibiting the activation of CD4^+^ T cells, and regulating the expression of Dnmt in CD4^+^ T cells.

## Materials and methods

### Animals

Male ApoE^−/−^ mice of 7 weeks old were purchased from Beijing Vital River Laboratory Animal Technology Co., Ltd. [certificate number: 11400700352760, permit number: SCXK (JING) 2016–0006]. All mice were housed and bred under specific pathogen-free (SPF) conditions of Nanjing University of Chinese Medicine Experimental Animal Center [permit number: SYXK (SU) 2014–0001]. All animal studies were approved by Nanjing University of Chinese Medicine Experimental Animal Center and were performed in strict accordance with “Principles for Use of Animals” and “Guide for the Care and Use of Laboratory Animals” of the U.S. National Institutes of Health.

### Animals treatment

Mice were departed into four groups as control, model (HFD + LPS), simvastatin and RES. Mice in control group were fed with normal diet, and HFD (1.25% cholesterol and 20% lard) were provided for 20 weeks to induce an advanced AS in mice of other groups. Besides, mice except for control group were attacked with 10 μg/mice of LPS (Sigma, L2880, 055:B5) i.p. once every 2 weeks for five times from the 10th week. 3.3 mg/kg (BW)/day of simvastatin (Merck Sharp & Dohme Ltd) and 5 mg/kg (BW)/day of RES dispersed in 5% sodium carboxymethyl cellulose solution were intragastric administrated daily, and mice in control group received same amount of 5% sodium carboxymethyl cellulose solution. RES (purity≥98%) was purchased from Shanghai yuanye Bio-Technology Co., Ltd. (CAS: 501–36-0).

### Serum lipids analysis

Peripheral blood of mice were collected from the posterior orbital venous plexus. The blood was centrifuged for 10 min under the rotate speed of 3000 rpm and the serum was collected for the analysis of serum lipids. The detection is performed according to the specification of enzymatic biochemical kits (Nanjing Jiancheng) via microplate reader (TECAN): Total Cholesterol Assay kit (Cat A111–1), Triacylglycerol Assay kit (Cat A110–1), Low Density Lipoprotein Cholesterol Assay kit (Cat A113–1), and High Density Lipoprotein Cholesterol Assay kit (Cat A112–1). The level of non-HDL-C was calculated by HDL-C subtracted from TC content.

### H&E staining of coronary artery

The hearts were fixed with 4% paraformaldehyde (PFA) and then embedded in paraffin. Further, they were sliced latitudinally, stained by hematoxylin-eosin (H&E), and finally shot by a digital microscope (magnification: × 400).

### Atherosclerotic plaque staining and analysis

Aortas were separated from the aortic root to the left and right common iliac artery and the redundant tissue outside the aortas was removed carefully under the microscope. Then the aortas were cut off vertically by microscopic scissors and were stapled on the black foam plate. Oil red O was used for aortas staining after 12 h fixation by 4% PFA and then 75% ethanol was used to differentiate normal tissue into creamy white. After thorough rinsing, the area of the atherosclerotic plaque was measured by MapInfo7.0 (USA, https://www.mapinfo.com).

### Cells assay

CD4^+^ T cells were sorted from the spleen of C57BL/6 mice according to the operating steps of Dynabeads Untouched Mouse CD4 Cells (Invitrogen, 11416D) and then adjusted to the density of 1 × 10^6^ cells/mL. Except for the control group, cells were stimulated by 1 μg/mL anti-CD3 (BioLegend, 100,202) and 1 μg/mL anti-CD28 (Biolegend, 102,102), and 0.1 μg/mL LPS were used to mimic an inflammatory environment for 24 h in 24-wells dishes. 20, 40, 80 μM of RES were added into cells and 1 μM of 5-Aza (MCE, Lot#28452) was pretreated for 36 h before the adding of LPS and RES in experiments necessary. CD4^+^ T cells were cultured in the cell incubator under the temperature of 37 °C and 5% CO_2_ and cultured by RPMI 1640 with 10% fetal calf serum (Gibco, New Zealand, 10,091,148) and 1% penicillin and streptomycin.

### Flow cytometry

For flow cytometry, fresh blood was collected in anticoagulant tubes (BD, 8215736) and peripheral blood mononuclear cells (PBMC) were obtained by lymphocyte isolate medium (KeyGEN BioTECH, kga831). Cells were washed and treated with the Fixation/Permeabilization Solution (BD) for Ki67 detection, incubated with flow cytometry antibodies (BD Pharmingen), and resuspended in phosphate-buffered saline (PBS) with 1% bovine serum albumin (BSA). Then cells were determined with BD FACS Calibur Flow Cytometer. Antibody collocations were as follows:

(i) CD4^+^ CD25^+^ T cells: FITC Anti-Mouse CD4 (eBioscience, clone: RM4–4), APC Anti-Mouse CD25 (eBioscience, clone: PC61.5);
(ii) CD4^+^ CD44^+^ T cells: FITC Anti-Mouse CD4 (eBioscience, clone: RM4–4), PerCP Anti-Mouse CD44 (BioLegend, clone: IM7);
(iii) CD4^+^ CD62L^+^ T cells: FITC Anti-Mouse CD4 (eBioscience, clone: RM4–4); PE Anti-Mouse CD62L (BD, clone: MEL-14);
(iv) CD4^+^ Ki67^+^ T cells: PE Anti-Mouse CD4 (BD, clone: RM4–5); FITC Anti-Mouse Ki67 (BioLegend, clone: 16A8).

### ELISA analysis

The supernatant from CD4^+^ T cells cultured above was subjected to detect the concentration of IL-2, IL-6, IL-10 and TGF-β1 using ELISA. The assay was conducted according to the procedures recommended in the manufacturer’s instructions of IL-2 (EK202HS-96), IL-6 (EK2061/2), IL-10 (EK210/3–96), TGF-β1 (EK2812/2) ELISA kits. All these kits were obtained from Multisciences (Lianke) BioTECH.

### Quantitative real-time PCR (qRT-PCR)

TRIzol Reagent (Invitrogen) was used to extract total RNA from spleens or aortas. 100 ng/10 μL of spleen or aorta RNA were quantified with NanoDrop One (Thermo Scientific) and 5× All-In-One RT MasterMix (abm) was used for reverse transcription in Veriti 96-Well Thermal Cycler (Applied Biosystems). 7500 Real-Time PCR (Applied Biosystems) was used for qRT-PCR using specific primers (synthesized by Sangon Biotech) and Eva Green 2× qPCR Master Mix-Low ROX (abm). Reverse transcription and amplification conditions followed the reagent instructions. Data were analyzed via the 2^-ΔΔCt^ method normalized to β-Actin. Sequences of primers were used as follows from 5′ to 3′ extremity:

(i) β-Actin: GGCTGTATTCCCCTCCATCG (F); CCAGTTGGTAACAATGCCATGT (R);
(ii) Dnmt1: ATCCTGTGAAAGAGAACCCTGT (F);
  CCGATGCGATAGGGCTCTG (R);
(iii) Dnmt3b: AGCGGGTATGAGGAGTGCAT (F);
  GGGAGCATCCTTCGTGTCTG (R)

### Western blotting (WB) analysis

Total protein was extracted from spleens or aortas by High Efficiency RIPA Lysis Buffer (KeyGEN BioTECH) and quantified by BCA protein concentration assay kit (Beyotime Biotechnology). Protein loading buffer (Beyotime Biotechnology) was added into protein and boiled for 5 min for protein degeneration. Thirty microgram of protein was loaded in 8% SDS-PAGE gel and transferred onto polyvinylidene fluoride (PVDF) membranes (Merck Millipore). Skim milk in TBST (TBS containing 0.1% Tween 20) was used as a blocking agent before antibody incubation. PVDF membranes were incubated with rabbit antibodies against Dnmt3b (Cell Signaling Technology) and GAPDH (Proteintech), mice antibody Dnmt1 (Abcam). Goat anti-rabbit IgG and goat anti-mice with HRP (Proteintech) were chosen as the secondary antibody. The imaging was performed in Gel Doc XR Biorad (Bio-Rad) using Chemiluminescent HRP Substrate (Merck Millipore). Data was analyzed with Image Lab 4.0 (Bio-Rad). Gray values of blot areas were measured, and the relative expression amount of the protein samples was calculated by the method of the target protein gray value/internal reference GAPDH gray value.

### Statistical analysis

Data are presented as the mean ± standard derivation (SD) and were analyzed with Prism 5.02 software (GraphPad). Statistical analysis was performed using the unpaired Student’s t-test to test the mean of two groups, and one-way analysis of variance (ANOVA) was applied for comparisons between multiple experimental groups. A value of *P* < 0.05 were considered statistically significant difference.

## Results

### RES regulated serum lipids and ameliorated AS in ApoE^−/−^ mice

**Fig. 1:**
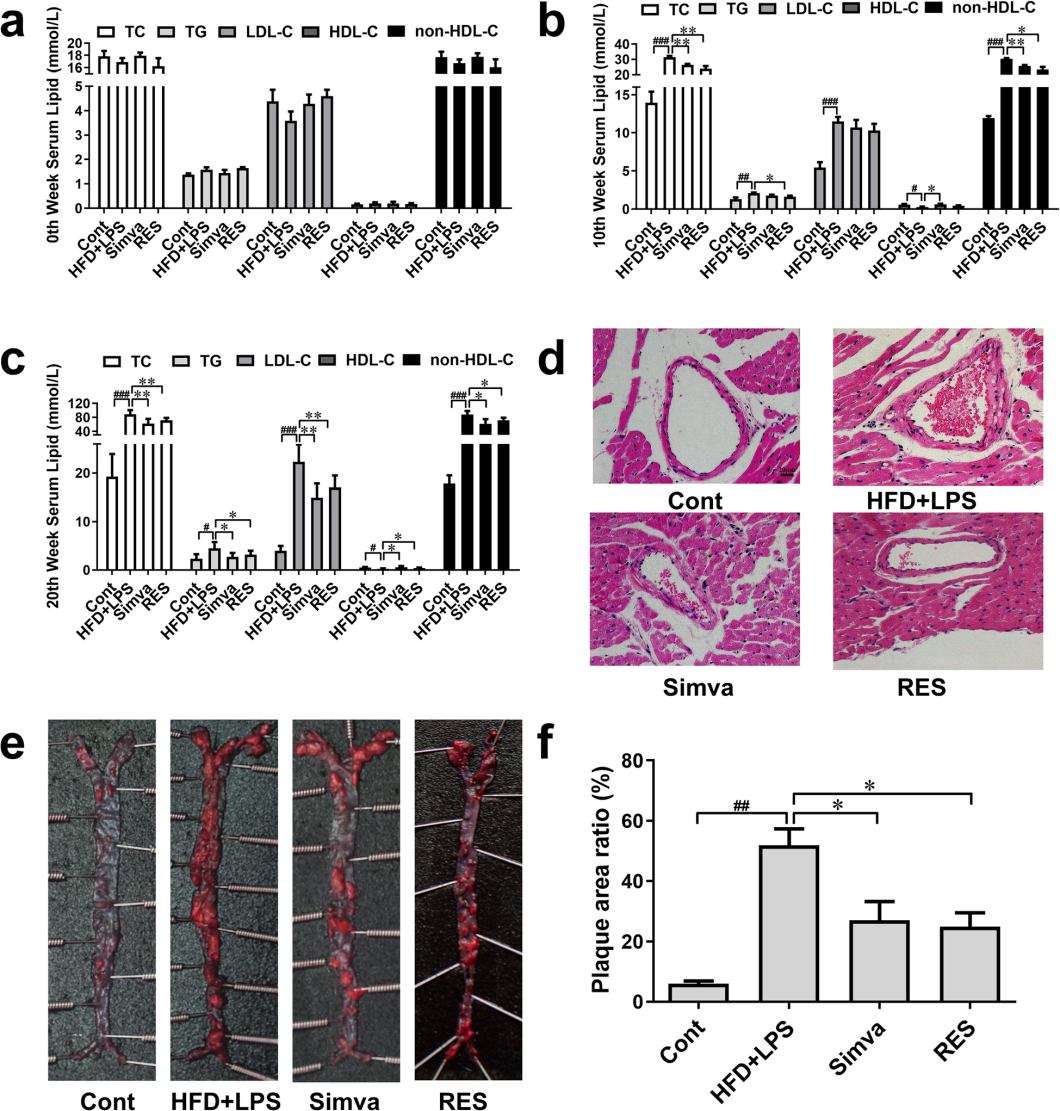
RES inhibited the progression of AS in vivo. Serum of mice was collected at 0th, 10th and 20th respectively and diluted by a quarter in saline and the lipids were detected according to the operating instruction of the kits. **a** The level of total cholesterol (TC), triglyceride (TG), low density lipoprotein cholesterol (LDL-C), high density lipoprotein cholesterol (HDL-C), and non-HDL-C of serum in the 0th week (*n* = 8). **b** The level of TC, TG, LDL-C, HDL-C, and non-HDL-C in the 10th week (*n* = 8). **c** The level of TC, TG, LDL-C, HDL-C, and non-HDL-C in the 20th week (*n* = 8). **d** Mice were killed at the end of the 20th week and the hearts were preserved. The pathologic condition of the coronary artery was analyzed by H&E. **e** The aortas were separated from the aortic arch to the left and right common iliac artery and were stained by Oil Red O. **f** The areas of aortic plaque were measured by MapInfo 7.0 (*n* = 5). Data are represented with mean ± SD, #*P* < 0.05, ##*P* < 0.01, ###*P* < 0.001 versus the control group; *P < 0.05, **P < 0.01 versus the HFD + LPS group

The level of serum lipids including TC, TG, LDL-C, and HDL-C in the 0th, 10th and 20th weeks of the experiment were detected while the content of non-HDL-C was calculated (Fig. 1a-c). The level of TC, TG, LDL-C, and non-HDL-C significantly increased after 10 weeks of HFD. At the 20th week, compared to mice of the control group, the concentration of TC, TG, LDL-C as well as non-HDL-C all increased while HDL-C level decreased. Compared to mice of HFD + LPS group, 5 mg/kg (BW)/day of RES decreased content of TC, TG and non-HDL-C at 10th week, and decreased content of TC, TG, LDL-C and non-HDL-C, meanwhile increased content of HDL-C at 20th week, suggesting that the ability of RES on regulating lipids in atherosclerotic mice induced by HFD and LPS will increase with prolonged use.

Both HFD and LPS are harmful to the mammal blood vessels. After 20 weeks of HFD together with five times of LPS injection, thickened coronary wall was observed (Fig. 1d), and large area of atherosclerotic plaque was detected on aortas (Fig. 1e-f). Simvastatin or RES treatment ameliorated the pathological change of coronary wall slightly. In addition, simvastatin or RES treatment decreased the infiltrated lesion of the aorta and the plaque area ratio significantly. These results indicated that RES treatment can inhibit the progression of AS.

### RES inhibited the activation of CD4^+^ T cells in atherosclerotic mice

**Fig. 2:**
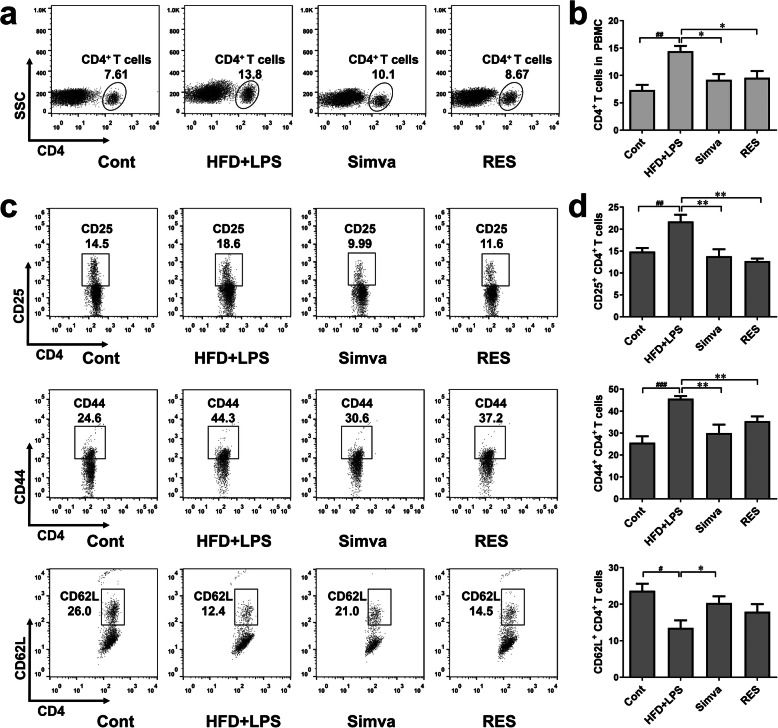
RES affected the frequency and activation of CD4^+^ T cells. Peripheral blood of mice was collected at the end of the 20th week and lymphocyte isolate medium was used to obtain peripheral blood mononuclear cells (PBMC). **a-b** CD4 FITC antibody was used and the frequency of CD4^+^ T cells in PBMC was detected by flow cytometry (*n* = 4). **c-d** CD4 FITC, CD25 APC, CD44 PerCP and CD62L PE antibody were used to stain the cells and the frequency of CD25^+^, CD44^+^ and CD62L^+^ cells in CD4^+^ T cells were analyzed by flow cytometry (*n* = 4). Data are represented with mean ± SD, #*P* < 0.05, ##*P* < 0.01, ###*P* < 0.001 versus the control group; *P < 0.05, **P < 0.01 versus the HFD + LPS group

In order to observe the effects of RES on CD4^+^ T cells in atherosclerotic mice, the frequency of CD4^+^ T cells in peripheral blood mononuclear cells (PBMC) was detected. It was observed that the frequency of CD4^+^ T cells was increased with combinational stimulation of HFD and LPS while RES decreased the ratio of CD4^+^ T cells in PBMC (Fig. 2a-b), which suggested RES improved the inflammatory status in AS. CD25, CD44 and CD62L were detected to analyze the activation of CD4^+^ T cells (Fig. 2c-d). It was found that HFD and LPS injection increased the expression of CD25 and CD44, and decreased CD62L expression in CD4^+^ T cells in peripheral blood. Intragastric administration of RES decreased CD25 and CD44 expression, but CD62L expression was not changed. The expression change of activation markers suggested that RES can reduce the expression of CD25 and CD44, but does not affect CD62L expression in AS mice with combinational stimulation of HFD and LPS.

### RES inhibited the activation of CD4^+^ T cells in vitro

**Fig. 3:**
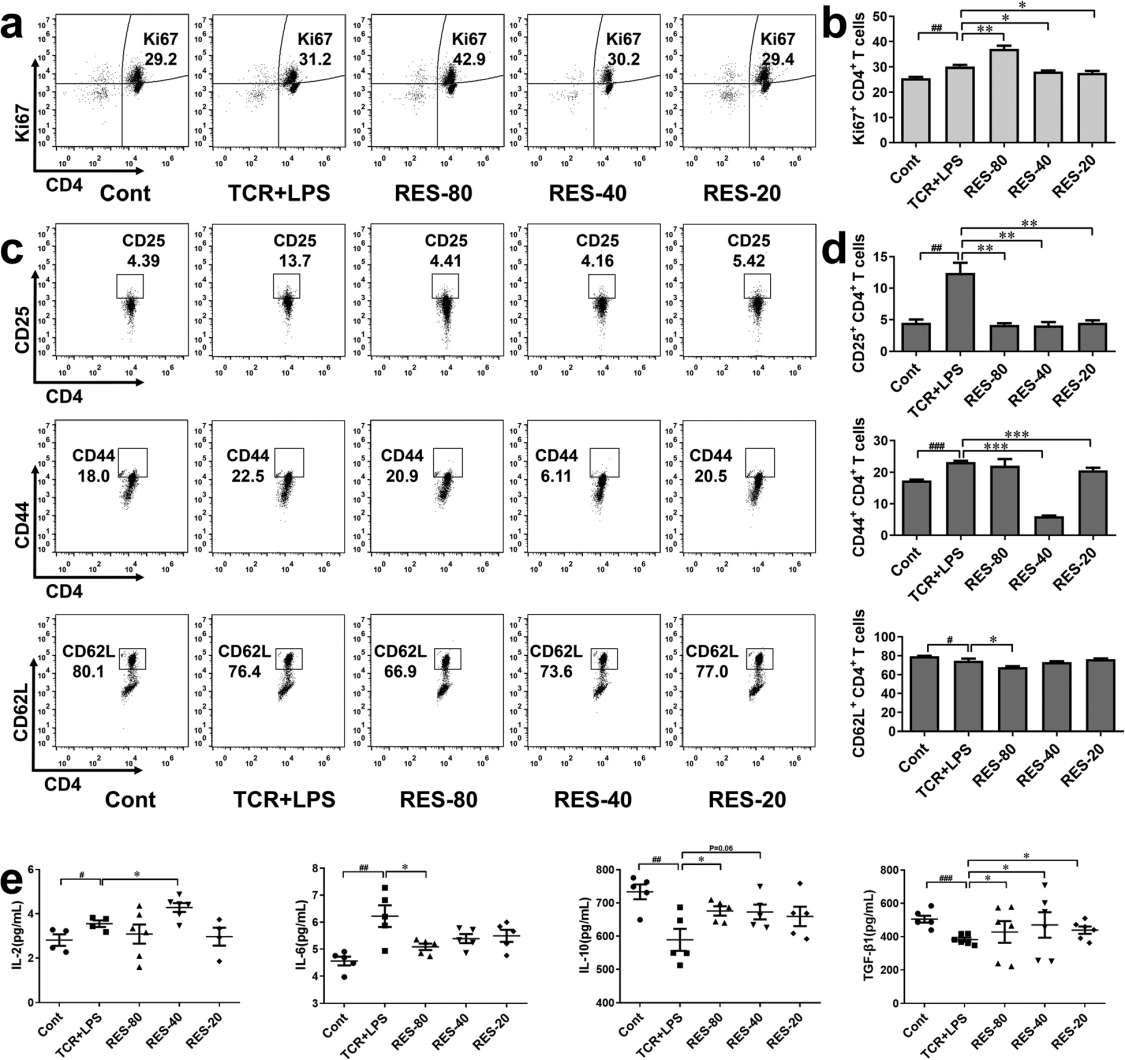
RES influenced the activation of CD4^+^ T cells in vitro. CD4^+^ T cells were stimulated by 1 μg/mL anti-CD3 and 1 μg/mL anti-CD28 and cultured in a cell incubator. 0.1 μg/mL LPS and 20, 40, 80 μM of RES were added and cells were collected 24 h later. **a-b** The expression of proliferation marker, Ki67 was observed by flow cytometry (*n* = 4). **c-d** The expression of activation markers including CD25, CD44, and CD62L of CD4^+^ T cells were observed by flow cytometry (*n* = 4). **e** The cytokines of IL-2, IL-6, IL-10, and TGF-β1 in the culture supernatant were detected by ELISA (*n* = 5). Data are represented with mean ± SD, #*P* < 0.05, ##*P* < 0.01, ###*P* < 0.001 versus the control group; *P < 0.05, **P < 0.01, ***P < 0.01 versus the TCR + LPS group

We further studied the effects of RES on the activation of CD4^+^ T cells in vitro. To access the proliferation of CD4^+^ T cells, Ki67, which is specifically expressed in proliferating cells, was detected (Fig. 3a-b). TCR and LPS treatment increased the expression of Ki67 in CD4^+^ T cells while 20 μM and 40 μM of RES decreased Ki67 expression under TCR and LPS treatment. Surprisingly, increased Ki67 expression was detected under 80 μM of RES treatment.

In addition, TCR and LPS stimulation significantly increased the expression of CD25 and CD44 and decreased CD62L expression in CD4^+^ T cells. 20, 40, and 80 μM of RES decreased the expression of CD25 in CD4^+^ T cells while 20 and 40 μM of RES decreased CD44 expression, the influence of 80 μM of RES on CD44 expression was not detected. It was notably that 20 and 40 μM of RES have no influence on CD62L expression, but 80 μM of RES unexpectedly decreased CD62L (Fig. 3c-d).

Cytokines secreted by CD4^+^ T cells were detected (Fig. 3e). 80 μM of RES increased IL-6, IL-10 and TGF-β1 level in supernatant but had no effect on IL-2 level. 40 μM of RES increased IL-2 and TGF-β1 but did not influence IL-6 and IL-10 secretion. 20 μM of RES increased the TGF-β1 level but did not change the secretion of IL-2, IL-6, and IL-10. RES was not powerful in regulating IL-2, IL-6, and IL-10 secretion, but promotes TGF-β1 secretion. In summary, different concentrations of RES has different effects on the expression of markers of proliferation and activation in CD4^+^ T cells as well as cytokines secretion.

### RES inhibited the expression of Dnmt1 and Dnmt3b in CD4^+^ T cells

**Fig. 4:**
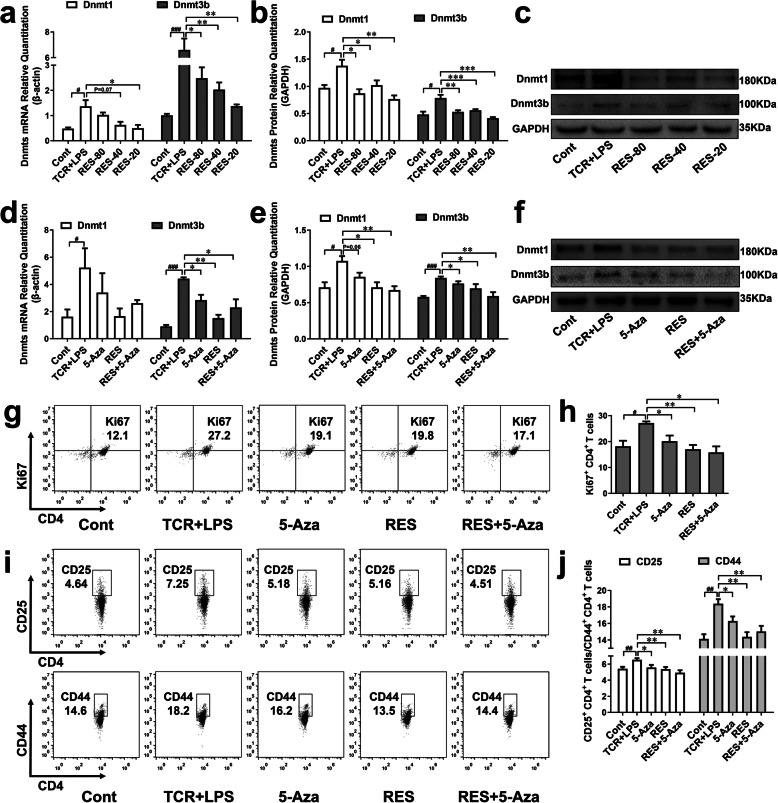
RES inhibited the expression of Dnmt1 and Dnmt3b in CD4^+^ T cells. CD4^+^ T cells were stimulated by 1 μg/mL anti-CD3 and 1 μg/mL anti-CD28 and cultured in a cell incubator. 0.1 μg/mL LPS and 20, 40, 80 μM of RES were added and cells were collected 24 h later. **a** mRNA level of Dnmt in CD4^+^ T cells detected by qRT-PCR (*n* = 4). **b-c** The protein level of Dnmt in CD4^+^ T cells detected by WB (*n* = 4). 5-Aza were pretreated for 36 h, and cells were collected after the treatment of LPS and 40 μM of RES. **d** mRNA level of Dnmt in CD4^+^ T cells (*n* = 4). **e-f** The protein level of Dnmt in CD4^+^ T cells (*n* = 4). **g-h** The expression of proliferation marker, Ki67 was observed by flow cytometry (*n* = 4). **i-j** The expression of activation markers including CD25 and CD44 of CD4^+^ T cells were observed by flow cytometry (*n* = 4). Data are represented with mean ± SD, #*P* < 0.05, ##*P* < 0.01, ###*P* < 0.001 versus the control group; *P < 0.05, **P < 0.01, ***P < 0.001 versus the TCR + LPS group

To observe the expression of Dnmt in CD4^+^ T cells, the level of mRNA and protein of Dnmt were detected (Fig. 4a-c). mRNA expression of Dnmt1 and Dnmt3b were increased in CD4^+^ T cells treated by TCR and LPS, and protein expression of Dnmt1 was also increased. 20 μM of RES decreased the expression of Dnmt1 and Dnmt3b in both mRNA and protein levels. 40 and 80 μM of RES decreased protein expression of Dnmt1 and decreased mRNA and protein expression of Dnmt3b. In order to confirm whether these expression changes of Dnmt related to the activities of CD4^+^ T cells, 5-Aza, an inhibitor for Dnmt was used. It was verified that 5-Aza significantly inhibited the expression of Dnmt3b in both mRNA and protein levels, but Dnmt1 is not influenced by 5-Aza (Fig. 4d-f). 40 μM of RES was similar to 5-Aza in inhibiting Dnmt3b and besides, 40 μM of RES decreased Dnmt1 protein expression, but mRNA expression of Dnmt1 has not been detected. Both 5-Aza and 40 μM of RES inhibited the expression of Ki67 (Fig. 4g-h), CD25 and CD44 (Fig. 4i-j). In addition, the combination of 5-Aza and 40 μM of RES reduced Dnmt1 protein level and Dnmt3b mRNA and protein level, and decreased the expression of Ki67, CD25, and CD44.

## Discussion

In our research, we demonstrated that RES effectively ameliorated AS induced by HFD companied with LPS in ApoE^−/−^ mice. In addition, our results indicated that RES treatment significantly inhibited the accumulation of lipids on vessel wall, inhibited the proliferation and activation of CD4^+^ T cells both in vivo and in vitro, and regulated the secretion of cytokines. Furthermore, we clarified that RES treatment inhibited the expression of Dnmt1 and Dnmt3b in CD4^+^ T cells, which related to their inhibited proliferation and activation.

AS is a chronic inflammatory disease [1]. High level of serum lipids and the inflammatory status are risk factors of AS [2, 27]. Feeding ApoE^−/−^ mice with HFD can induce AS and LPS injection can accelerate the progression of disease [28]. In our study, HFD companied with LPS induced dysfunction of serum lipids and formation of atherosclerotic plaque. Administration of RES counteracts thickened coronary artery, decreased the accumulation of lipids on the aortic wall, and regulated the level of serum lipids, indicated the positive function of RES on HFD and LPS induced AS in ApoE^−/−^ mice.

Dyslipidemia is a recognized risk factor of AS. In our study, RES decreased the high level of TC and TG in 10th week of the experiment and inhibited the dysfunction of TC, TG, LDL-C as well as HDL-C in 20th week. RES has been widely reported to regulate serum lipids [29, 30], promote the degradation of ox-LDL [31], suppress adipocyte differentiation and TC accumulation, and stimulate lipolysis. RES powerfully regulated the serum lipids and our result further verified this efficiency and hinted that RES play this function in a long time.

Rather than regulated serum lipids, RES attenuated Trimethylamine-N-Oxide (TMAO)-induced AS in ApoE^−/−^ mice [32], ameliorated HFD induced AS in LDLR^−/−^ mice [33], countered systemic lupus erythematosus-associated AS [34] and alleviated AS in atherosclerotic mice whose left carotid artery was partial ligated [35]. Our result is consistent with previous studies that RES possess beneficial effects on AS and the influences of RES on AS induced by HFD and LPS was firstly observed in our research. LPS is an inflammatory risk factor. The infection of Gram-negative bacterial can increase the level of LPS [36]. Elevated LPS is easily to induce a low-grade inflammation state, which is another risk factor for AS in addition to a high level of serum lipids [37].

RES ameliorates the symptoms of AS through multiple mechanisms. RES regulates the production of vasodilator and vasoconstrictor, inhibits the generation of oxidative stress/reactive oxygen species and ameliorates inflammation [38]. But the precise mechanism of how RES alleviate AS still complex and incompletely clarified. Our study clarified that inhibiting the activation of CD4^+^ T cells is also a mechanism of RES in ameliorating AS, and the down-regulated Dnmt1 and Dnmt3b in CD4^+^ T cells induced by RES participated in the inhibited activation of CD4^+^ T cells.

CD4^+^ T cells are the most abundant T cells population in atherosclerotic plaque and play important roles throughout all stages of AS. CD62L is critical for migration of naïve CD4^+^ T cells into lymphoid tissue and highly expressed on naïve CD4^+^ T cells and downregulated upon the activation [39]. Besides, CD4^+^ T cells increase the expression of CD25 and CD44 after activation [40–43].

Activated CD4^+^ T cells response to the antigen signal, secret inflammatory cytokines and began to proliferation. Increased and activated CD4^+^ T cells accelerate the progression of inflammation and induce the progression of AS [15]. RES inhibited the proliferation and activation of CD4^+^ T cells both in atherosclerotic mice and in cultured splenic CD4^+^ T cells in vitro, which remained us that RES ameliorated AS by inhibiting the proliferation and activation of CD4^+^ T cells.

CD4^+^ T cells are constituents of the adaptive immune response [44]. Activated CD4^+^ T cells secrete proinflammatory cytokines and inflammation mediated by activated CD4^+^ T cells plays an important role in the initiation and progression of AS [45]. RES decreased the secretion of IL-6, increased IL-10 and TGF-β1. IL-6 contributes to host defense through the stimulation of acute phase responses, hematopoiesis, and immune reactions. Continual secretion of IL-6 plays a pathological effect on chronic inflammation [46]. It has been reported that low content of IL-10-producing CD4^+^ T cells is a risk factor for progression of coronary AS [47] and evoking TGF-β1-mediated anti-inflammatory response inhibited AS progression [48]. Besides, RES increased the secretion of IL-2 in CD4^+^ T cells. IL-2 is a proinflammatory cytokine and the level of IL-2 may be close correlated with the development and progression of CVD [49, 50]. But IL-2 can induce the development of Tregs, which protect against AS [51]. The effects of increased IL-2 in AS still under discussion. RES promoted lymphocyte proliferation and IL-2 production [52] and we confirmed the same function of RES on CD4^+^ T cells. RES inhibited the activation and proliferation of CD4^+^ T cells, regulated the secretion of cytokines. We concluded that RES restrained the immune response of CD4^+^ T cells.

Researches about RES are various as RES can modulate numerous targets and molecular functions, and has various of biological activities including anti-inflammatory, anti-oxidation, prevent and treatment of cardiovascular diseases, and anti-cancer. Animal experiments and clinical researches about RES are numerous for its moderate effects on various pathological conditions. RES can ameliorate AS in animal studies, RES decrease TC, TG, LDL-C, and increase HDL-C by regulating the level of the hepatic 3-hydroxy 3-methylglutaryl coenzyme A (HMG-CoA) reductase enzyme or cholesterol 7α-hydroxylase (CYP7A1) [53, 54]. The anti-oxidative function of decreasing the formation of ox-LDL of RES is also beneficial for the prevention of AS [55]. Anti-inflammatory properties can be regarded as a part of anti-atherosclerotic effects of RES as inflammation is essential in the progression of AS [56]. RES inhibit the secretion of inflammatory cytokines including IL-1β, IL-6, and TNF-α and inhibit the inflammatory signal transduction such as NF-κB signaling [57, 58]. In addition, RES ameliorates AS by inhibiting platelet aggregation and inducing platelet apoptosis [59, 60]. Generally, we can conclude that RES treat AS from all stage of the progression in preclinical researches.

The anti-atherosclerotic function of RES in animal studies is promising, but the clinical trials are still limited. As for the plasma lipids, the effects of RES on healthy adults or patients with CVDs are not consistent [61, 62]. The small size and heterogeneity of the study populations, heterogeneous study designs, the dose of RES, and duration of RES supplementation make the clinical researches of RES on AS treatment limited and the anti-atherosclerotic effects of RES call for further well-controlled clinical trials that assess the effect of RES on all the aforementioned steps in the atherosclerotic process besides of the plasma lipid profile [63].

The level of CD25, CD44, and CD62L, the secretion of IL-2, and the expression of Dnmt1 and Dnmt3b of CD4^+^ T cells were not observed to be dose dependent with RES treatment in our research. Marco Craveiro et al. reported that 20 and 100 μM of RES possess different influences on human CD4^+^ T cells stimulated by TCR [21]. Both 20 and 100 μM of RES decreased the expression of CD25 and CD71 but did not affect CD69 expression. In addition, 20 μM of RES inhibited the proliferation of human CD4^+^ T cells while 100 μM of RES had no effects. Our results together with these results indicated that the different concentration of RES can cause different or even opposite effects on activation and proliferation of CD4^+^ T cells, which remained us that the dose of RES in application should pay enough attention. Changed physiological activities induced by RES may achieved by multiple ways because RES possesses wide biological activities as listed above and different dose of RES produce different effects may due to this. Many researches pointed that the dose should be noted in the use of RES. At present, RES has been widely applicated as health products for the positive effects on human health. The wide biological activities of RES are the basis of RES to ameliorate multiple diseases, but the low specificity may also obstructed the development of medicinal value and clinical application of RES. The exact mechanism of RES should be clarified.

Dnmt1 maintain the methylation and Dnmt3b mediate the de novo methylation. Aberrant or abnormal expression of Dnmt is associated with a variety of human diseases including AS. Therefore, Dnmt are promising therapeutic epigenetic targets as specific inhibitors might regulate the expression of Dnmt and stop or even reverse aberrant cellular processes [64, 65]. RES has been described as a regulator of Dnmt expression and majority of the research concluded that RES function as inhibitors of Dnmt in many kind of cells [66]. In our research, RES inhibited the increased Dnmt1 and Dnmt3b induced by anti-CD3/CD28 companied with LPS in CD4^+^ T cells, which is consistent with the research that RES inhibited Dnmt expression. In addition, the similar function of RES and 5-Aza on inhibiting the activation of CD4^+^ T cells remind us that down-regulated expression of Dnmt induced by RES may related to the inhibited activation of CD4^+^ T cells.

There are some new sights in this research that our study suggested RES ameliorated AS induced by HFD together with LPS in ApoE^−/−^ mice. In addition, RES inhibited the proliferation and activation as well as regulated the secretion of cytokines in CD4^+^ T cells. Finally, RES function like an inhibitor of Dnmt1 and Dnmt3b in CD4^+^ T cells but the inner mechanisms of how RES decreased the expression of Dnmt1 and Dnmt3b have not been clarified and we think it is meaningful to explore it.

Limitations also existed in this research. We investigated that RES can inhibit the expression of Dnmt1 and Dnmt3b in CD4^+^ T cells and clarified that the decreased expression of Dnmt1 and Dnmt3b related to the inhibited activation of CD4^+^ T cells, but whether RES inhibited the activation of CD4^+^ T cells through down-regulating Dnmt1 and Dnmt3b remains unclear.

## Conclusion

In summary, our research demonstrated that RES administration ameliorated AS induced by HFD companied with LPS in ApoE^−/−^ mice, inhibited the activation of CD4^+^ T cells, and down-regulated the expression of Dnmt1 and Dnmt3b in CD4^+^ T cells. Besides, the changed expression of Dnmt in CD4^+^ T cells is related to their proliferation and activation. This work clarified that inhibit the proliferation and activation of CD4^+^ T cells is one of the mechanism of RES on ameliorating AS and firstly investigated the decreased expression of Dnmt1 and Dnmt3b in CD4^+^ T cells induced by RES, and provided more evidences for the application of RES.

## Data Availability

The datasets used and/or analysed during the current study are available from the corresponding author on reasonable request.

## Abbreviations

AS: Atherosclerosis
CD62L: L-selectin
Cont: Control
CVD: Cardiovascular disease
Dnmt: DNA methyltransferase
HDL-C: High density lipoprotein cholesterol
HFD: High-fat diet
IL: Interleukin
LDL-C: Low density lipoprotein cholesterol
PBMC: Peripheral blood mononuclear cells
PFA: Paraformaldehyde
RES: Resveratrol
Simva: Simvastatin
TC: Total cholesterol
TG: Triglyceride
TGF-β1: Transforming growth factor-β1
5-Aza: 5-Azacytidine
